# Different Evolutionary History for Basque Diaspora Populations in USA and Argentina Unveiled by Mitochondrial DNA Analysis

**DOI:** 10.1371/journal.pone.0144919

**Published:** 2015-12-14

**Authors:** Miriam Baeta, Carolina Núñez, Sergio Cardoso, Leire Palencia-Madrid, Sergio Piñeiro-Hermida, Miren Arriba-Barredo, María Jesús Villanueva-Millán, Marian M. de Pancorbo

**Affiliations:** BIOMICs Research Group, Centro de Investigación “Lascaray” Ikergunea, Universidad del País Vasco UPV/EHU, Vitoria-Gasteiz, Spain; University of Florence, ITALY

## Abstract

The Basque Diaspora in Western USA and Argentina represents two populations which have maintained strong Basque cultural and social roots in a completely different geographic context. Hence, they provide an exceptional opportunity to study the maternal genetic legacy from the ancestral Basque population and assess the degree of genetic introgression from the host populations in two of the largest Basque communities outside the Basque Country. For this purpose, we analyzed the complete mitochondrial DNA control region of Basque descendants living in Western USA (n = 175) and in Argentina (n = 194). The Diaspora populations studied here displayed a genetic diversity in their European maternal input which was similar to that of the Basque source populations, indicating that not important founder effects would have occurred. Actually, the genetic legacy of the Basque population still prevailed in their present-day maternal pools, by means of a haplogroup distribution similar to the source population characterized by the presence of autochthonous Basque lineages, such as U5b1f1a and J1c5c1. However, introgression of non-Basque lineages, mostly Native American, has been observed in the Diaspora populations, particularly in Argentina, where the quick assimilation of the newcomers would have favored a wider admixture with host populations. In contrast, a longer isolation of the Diaspora groups in USA, because of language and cultural differences, would have limited the introgression of local lineages. This study reveals important differences in the maternal evolutionary histories of these Basque Diaspora populations, which have to be taken into consideration in forensic and medical genetic studies.

## Introduction

**Fig 1:**
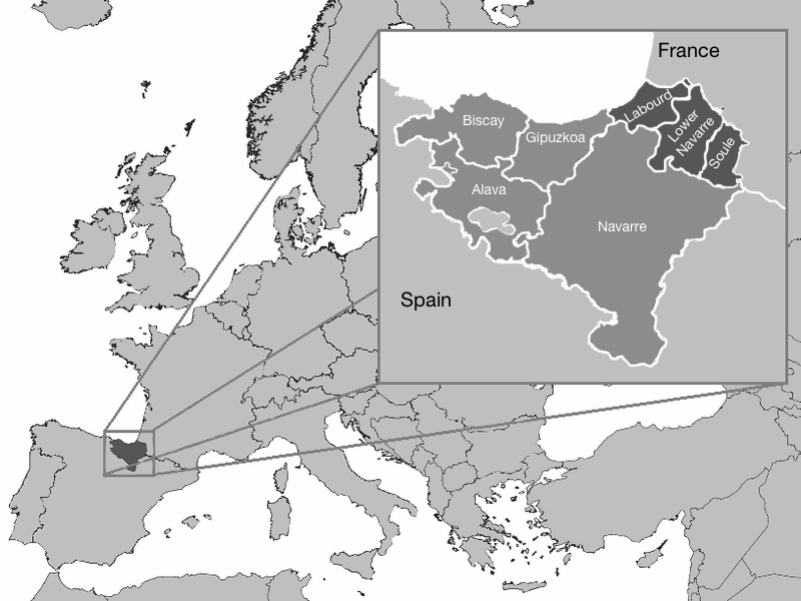
Map showing the location of the Basque territory in northern Spain and southern France. The provinces of Alava, Biscay, Gipuzkoa, and the Chartered Community of Navarre in Spain, and the provinces of Labourd, Lower Navarre, and Soule in France are indicated. Adapted from Wikimedia Commons (https://commons.wikimedia.org/wiki/File:Euskal_Herria_Europa.png); photo of Euskal Herria Europa by Zorion, under the Creative Commons Attribution License (CCAL) CC BY 3.0.

The Basque population has been traditionally considered as an isolate group in Europe because of its unique language (*Euskera*), geographical location, cultural tradition and genetic structure [1–3]. The cultural Basque-speaking territory, also known as *Euskal Herria*, encompass a region at both sides of the Atlantic Pyrenees: the Basque Country (Alava, Biscay and Gipuzkoa) and Navarre in Spain, and Lower Navarre, Labourd, and Soule in France (Fig 1).

During centuries, different socio-economic or political reasons have forced Basques to leave their ancestral territories. Nowadays, nearly 10 million descendants of Basque emigrants are estimated to be settled around the world, most of them preserving their identity, language and culture [4].

The Basque community in USA is one of the most numerous groups of Basque Diaspora outside of Europe. More than 57,000 individuals identified themselves as Basque Americans in the 2000 US Census, with an especially notable presence in the Western States of California, Nevada and Idaho. Although Basque arrival to America dates back to the Spanish colonial period of the 15th and 16th centuries and to the religion missions in the 17th century, the major migratory influx to North America was because of the Gold Rush in the middle of 18th century [5]. Furthermore, Spanish speaking American countries have also been important settling points for Basque emigrants since the middle of the 19th century, in particular the southern core of South America (Argentina, Uruguay, and Chile). In fact, nowadays Argentina presents, by far, the largest Basque Diaspora in the world, with more than 3 million people claiming to be Basque descendants (source: euskosare.org). In this case, the knowledge of Spanish language by Basques was one of the obvious advantages for their rapid adaptation and integration into the local cultures.

The flow of migrants has remained irregular until recent times. The settlement of newcomers was facilitated by existing infrastructures (Basque Houses or Euskal Etxeak), which helped with housing and employment [6, 7].

Autochthonous Basque population from northern Spain has been extensively subjected to genetic studies because of their genetic singularity [8–10]. In particular, mitochondrial DNA (mtDNA) analyses have revealed the low gene diversity of this population as well as the presence of specific haplogroups [3, 11, 12]. Besides, the genetic introgression from other Spanish maternal lineages in the genetic pool of Basques seems to have been really limited because of the historical isolation of the Basque population. French Basque population seems to share common features in the mtDNA pool with the Basques from northern Spain, although a certain degree of genetic differentiation has been described, which may be explained by genetic drift or different admixture influences [13]. So far, few genetic studies have focused on Basque Diaspora populations and the contribution of the host populations to their genetic legacy [14–17].

In order to study the genetic structure and the degree of introgression of non-Basque groups in Diaspora populations, here we present the analysis of the complete mtDNA control region of two of the largest Basque communities outside the Basque Country: the Basque Diaspora populations in Western USA and Argentina.

## Materials and Methods

### Sample collection

Mouthwash samples were obtained from 369 self-identified Basque descendants from two Diaspora populations (Western USA and Argentina). The population sample from the Basque Diaspora in the Western USA includes 175 individuals living in Boise (Idaho), Chino (California), and Reno (Nevada). On the other hand, the population sample from the Basque Diaspora in Argentina comprises 194 individuals living in the Buenos Aires province. All participants provided their written informed consent, and were asked for demographic and familial information. The followed procedures were in accordance with the ethical standards of the Helsinki Declaration. This study was approved with the favorable ethical report from the Faculty of Pharmacy of the University of the Basque Country, signed at 26th September 2008.

Both populations were classified in two groups: 1) DDV (non-admixed) group includes individuals with ancestors’ surnames that support maternal autochthonous Basque ancestry for at least three generations (81 in USA and 72 in Argentina), and 2) DDA (admixed) corresponds to those individuals who descend from Basques but have not maintained their maternal Basque surnames (94 in USA and 122 in Argentina). The criteria for this classification were based on the information collected from each donor.

### DNA extraction and quantification

DNA was extracted using the Gentra^®^ Puregene^®^ Kit from Buccal Cells in mouthwash (Gentra Systems, MN, USA). DNA was quantified with the Quant-iT PicoGreen dsDNA Assay Kit (Invitrogen, Carlsbad, CA) in a DTX880 Multimode Detector (Beckman Coulter, Fullerton, CA) and diluted to 10 ng/μL.

### PCR amplification and sequencing analysis

The entire mitochondrial DNA control region was amplified and sequenced as in [18]. The mtDNA control region sequences were edited between 16024 and 576 positions using the ChromasPro v3.1.0.0 (Technelysium Pty Ltd, Tewantin, QLD**,** Australia). Sequences were aligned and compared with the revised Cambridge reference sequence [19] using Clustal X v2.0 [20] and checked with SeqScape^®^ software v2.5 (Applied Biosystems, Foster City, CA, USA).

### Statistical analysis

Haplotypes were classified into haplogroups using the online tool Haplogrep [21] and manually confirmed following the updated version of the mitochondrial DNA phylogeny (www.phylotree.org, v16) [22].

Diversity parameters and inter-population Fst comparisons, based on mtDNA haplotypes were calculated using Arlequin v3.5.1.2 software [23]. For Fst comparisons, two European Basque-speaking source populations were included, one from northern Spain (N = 206), which integrated the Basque Country (Alava, Biscay, and Gipuzkoa provinces) [24] and North Navarre (GenBank numbers: JX294751-JX294850), and another from the French Basque Country (N = 193) (Lapurdi/Baztan, Lapurdi Nafarroa, and Zuberoa) [25]. The length polymorphisms of the poly-C stretch at HVS-I, HVS-II and HVS-III were disregarded in the analyses.

Phylogenetic comparisons were visualized with a multidimensional scaling (MDS) analysis using SPSS Statistics v18.0.0 (http://www.spss.com.hk/statistics/), based on the relative haplogroup frequency of the Basque Diaspora populations and other worldwide populations (S1 Table). Additionally, a Principal Component Analysis (PCA) was performed using PAST software v.3.04 [26], including the studied groups and European populations.

All sequences were deposited into EMPOP under accession numbers (USA: EMP00366 and EMP00367, and Argentina: EMP00664 and EMP00665). Likewise, these sequences from Western USA and Argentina are also available online at GenBank under accession numbers HQ200004-HQ200179 and KP784456-KP784649, respectively.

## Results

### Diversity of the maternal lineages

The complete mtDNA control region has been studied for the first time in the Basque Diaspora population from the Western USA and Argentina. The Western USA Basque Diaspora was formed by 175 individuals from maternal Basque ancestry (USA-DDV n = 81 and USA-DDA n = 94) and Argentinean Basque Diaspora by 194 individuals (ARG-DDV n = 72 and ARG-DDA n = 122) (S2 Table). The results indicated that the most frequent haplotypes identified in Diaspora groups are among the most represented in the Basque populations (S3 Table).

The diversity indices, e.g. haplotype (H) and nucleotide diversity (π_n_) indices and mean nucleotide pairwise differences (π), for each Basque Diaspora group are shown in Table 1. The results revealed that, in Western USA, the DDV group showed lower diversity values than DDA, whereas both Diaspora groups of Argentina presented similar values. Besides, the genetic diversity observed in these groups was similar or superior to the observed in the source Basque populations from Spain (H = 0.9812±0.0032) [24] and France (H = 0.9661±0.0073) [25].

**Table 1:** Diversity indices in Basque Diaspora populations.

	**USA**		**ARGENTINA**	
	USA-DDV (n **=** 81)	USA-DDA (n = 94)	ARG-DDV (n = 72)	ARG-DDA (n = 122)
Number of haplotypes	52	76	65	93
Number of unique haplotypes	39	62	62	79
Number of polymorphic sites	103	129	137	161
Haplotype diversity (H)	0.9765±0.0080	0.9943±0.0029	0.9945±0.0044	0.9904±0.0037
Mean number of pairwise differences (π)	8.7287±4.0705	10.1082±4.6597	12.1358±5.5488	11.7976±5.3770
Nucleotide diversity (π_n_)	0.0077±0.0040	0.0090±0.0046	0.0107±0.0054	0.0104±00527

Populations: Basque Diaspora populations in Western USA (USA-DDV and USA-DDA) and in Argentina (ARG-DDV and ARG-DDA). Range of mtDNA bases included: 16024–576.

In order to determine the existence of significant differences between these groups, Fst genetic distances based on haplotypes (and their corresponding p-values) were calculated (Table 2). In this regard, Diaspora groups from Western USA did not show statistically significant differences among them (Fst = 0.0045, p = 0.0889±0.0030). Likewise, no significant differences were observed between the USA-DDV group and the source Basque populations of Spain (Fst = -0.0012, p = 0.6023±0.0047), and France (Fst = 0.0036, p = 0.1249±0.0034). Contrarily, the USA-DDA group showed substructure from the two source groups (Spanish Basques: Fst = 0.0154, p = 0.0001±0.0001 and French Basques Fst = 0.0251, p<0.0001). Furthermore, no significant p-values were found among the two Argentinean Diaspora groups (Fst = -0.0018 p = 0.6635±0.0052), but both showed genetic substructure from the source Basque populations of Spain (ARG-DDV: Fst = 0.0262 p<0.0001 and ARG-DDA: Fst = 0.0149 p<0.0001) and France (ARG-DDV: Fst = 0.0311 p<0.0001 and ARG-DDA: Fst = 0.0169 p<0.0001). Finally, this analysis also highlighted the statistically significant differences between Diaspora groups from Western USA and the groups from Argentina.

**Table 2:** Genetic distances based on Fst (below the diagonal) with the corresponding p-value (over the diagonal).

	USA-DDV	USA-DDA	ARG-DDV	ARG-DDA	BAS	BAF
USA-DDV	*	**0.0889±0.0030**	0.0002±0.0001	0.0078±0.0008	**0.6023±0.0047**	**0.1249±0.0034**
USA-DDA	**0.0045**	*	0.0177±0.0013	0.0040±0.0006	0.0001±0.0001	0.0000±0.0000
ARG-DDV	0.0201	0.0085	*	**0.6635±0.0052**	0.0000±0.0000	0.0000±0.0000
ARG-DDA	0.0103	0.0088	**-0.0018**	*	0.0000±0.0000	0.0000±0.0000
BAS	**-0.0012**	0.0154	0.0262	0.0149	*	**0.2514±0.0040**
BAF	**0.0036**	0.0251	0.0311	0.0169	**0.0009**	*

Non-significant Fst values (p≥0.05) and the corresponding p-value are indicated by bold type. Populations: Basque Diaspora populations in Western USA (USA-DDV and USA-DDA) and in Argentina (ARG-DDV and ARG-DDA), autochthonous Basque population from Spain (BAS) and French Basque Country population (BAF).

### Ancestry of the maternal lineages

Haplogroup frequencies of the Diaspora populations from Western USA and Argentina are showed in S4 Table.

**Fig 2:**
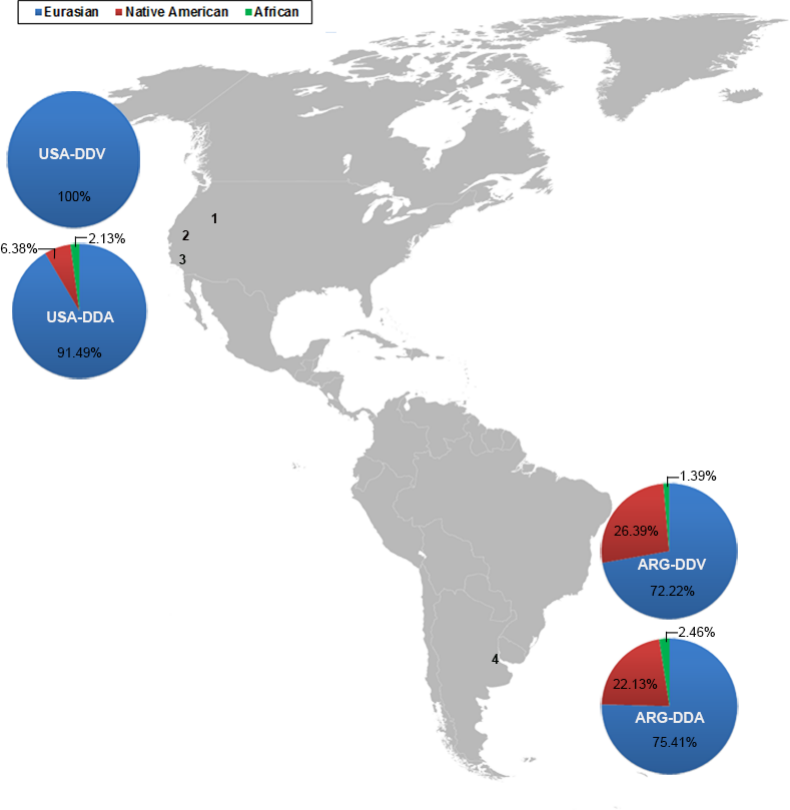
Distribution of Eurasian, Native American and African lineages in the Basque Diaspora populations. Geographical origin of samples is indicated by a number, Boise (1), Reno (2), and Chino (3) from USA, and Buenos Aires province (4) from Argentina. Adapted from Wikimedia Commons (https://commons.wikimedia.org/wiki/File:America-blank-map-01.svg); photo of Blank Politic Map of Americas by DavoO, under the Creative Commons Attribution License (CCAL) CC BY 3.0.

Noticeable differences in haplogroup composition were found in the Diaspora groups of Western USA. Remarkably, in DDV group, all the haplogroups had Eurasian phylogeographic origin (Fig 2) [27]. The most common maternal lineage was R0 (50.61%) (excluding cluster HV0), as similarly occurs in most West Eurasian populations [28, 29]. The polymorphisms within the mitochondrial control region do not allow resolving the phylogeny of this clade in detail, even though 18.52% of the individuals could be assigned to H subhaplogroups. Furthermore, it was possible to detect other haplogroups commonly found in other European populations, namely U (16.05%), J (8.64%), K (8.64%), T (6.17%), X (6.17%), V (2.47%) and I (1.23%).

The DDA group from Western USA, despite the dominant presence of Eurasian mtDNA lineages (91.49%), also presented minor Native American (6.38%) and African (2.13%) contributions. Among the Eurasian lineages, the main haplogroup was R0 (46.81%) (excluding cluster HV0), followed by T (9.57%) and U (8.51%). Concerning the Native American haplogroups, all four major founding haplogroups were detected (A, B, C and D) [30]. The most frequent one was A2, with a frequency of 2.12% in the DDA sample and 33.33% among the Native American haplogroups. Two African haplogroups, L2a and L3h, were also found (1.06% each) [31, 32].

In Argentina, the DDV group presented a predominance of Eurasian mtDNA lineages with a frequency of 72.22%. The diversity of haplotypes of Eurasian origin was high (0.9902±0.0080), being R0 the most common haplogroup in the population (25%, excluding HV0), followed by U (12.50%) and J (8.33%). This population presented a substantially contribution of Native American lineages, with a value of 26.39%, being the lineage B4b (11.11%) the most frequent one. A minimal African component was detected, more particularly one individual belonging to L3f1b1a.

The ARG-DDA group also presented a trihybrid structure with a major Eurasian component (75.41%), followed by Native American (22.13%) and African (2.46%) components. Haplogroup R0 was the most frequent lineage, representing 33.61% of the DDA sample (excluding HV0) and 44.57% of all Eurasian haplogroups. The second most abundant haplogroup was U with a frequency of 14.75%. Among the Native American lineages, the two most common belonged to A2 and B4b haplogroups, with a 5.74 and 4.92%, respectively. On the other hand, African lineages corresponded to L3h (1.64%) and L1c (0.82%).

#### Basque genetic signature in Diaspora populations

The maternal Basque contribution to the Diaspora groups here studied is well-established by the presence of autochthonous lineages from the Basque Country, namely U5b1f1a and J1c5c1 [3]. In this regard, U5b1f1a was detected in all Diaspora groups, but in a high percentage in the USA-DDV (11.11%) and Argentinean groups (ARG-DDA: 9.02% and ARG-DDV: 4.17%). On the other hand, although the lineage J1c5c1 was solely found in lower frequencies in these three groups (1.39–2.47%), the major haplogroup J was present in a notorious percentage in the four sample sets (5.32–8.64%), close to the 9–11% of the Basque samples [11, 24]. In the present study, it was not possible to define the V22 lineage, also considered as an autochthonous Basque marker [3], because of the lack of defining mutations in the control region. However, a percentage of 1.39–2.50% of lineages in the four Diaspora groups belonged to haplogroup V19’22, being potentially subsumed as V22. Other lineages, i.e. H1 and H3, have also been considered as potential Basque lineages, as they present elevated frequencies in Basque populations in northern Spain (H1: ~15% and H3: ~5%). In the present study, based on the control region analysis, lineages belonging to H1 were more frequent (2.78–7.50%) than H3 (0–2.50%) [12]. Moreover, recent studies have deepened in the phylogeny of this clade, identifying the variants of H lineages H1j1, H1t1, H2a5a1, H1av1, H3c2a, and H1e1a1 [25], or HV4a1a [33] as originated from the Basque region. Of these mtDNA variants, our analysis of the control region allowed the identification of H1t1 in the Diaspora populations. Another subhaplogroup described as a Basque Paleolithic marker is U8a, which presents a 1% frequency in the Basque Country [34]. However, other authors postulated about a Mediterranean origin for this haplogroup, as a higher percentage has been detected in that area (8.10%) [35]. In the present study, the presence of this variant has been rare, only observed in three individuals.

#### Phylogeography of the European lineages of the Basque Diaspora

**Fig 3:**
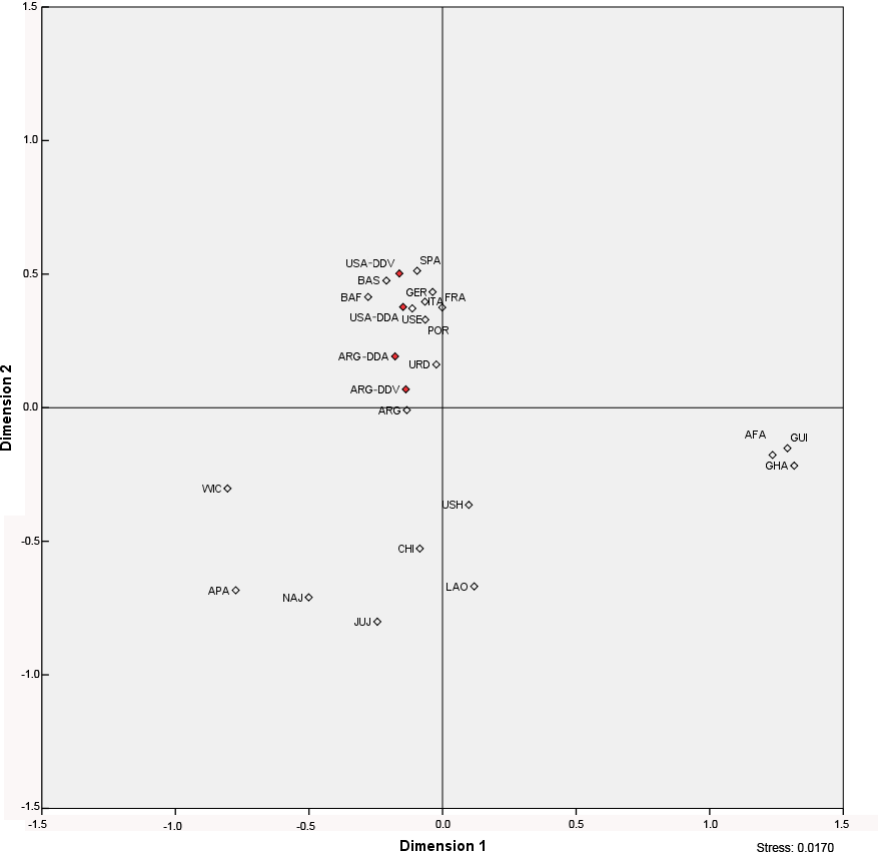
MDS showing distances between Basque Diaspora populations and other populations, based on mtDNA haplogroup frequencies. Populations included are as follows: Basque Diaspora populations (in red) from USA (USA-DDV and USA-DDA) and Argentina (ARG-DDV and ARG-DDA); Basque populations from Spain (BAS) and France (BAF); France (FRA); Germany (GER); Italy (ITA); Portugal (POR); Spain (non-Basque included) (SPA); African American (AFA), Apaches (APA), Navajos (NAJ), Hispanos (USH), and European descents (USE) from USA; Jujuy (JUJ), and residents (ARG) from Argentina; Basque Diaspora from Uruguay (URD); South-West China (CHI); Laos (LAO); Guinea-Bissau (GUI); and Ghana (GHA).

To determine the genetic affinity of the Basque Diaspora populations in a global context, a MDS analysis, based on haplogroup frequencies, was performed (Fig 3). All the populations with a high European genetic background, including Basque Diaspora groups, were grouped in the plot, well differentiated from African, Hispanic, Asiatic and Native American populations. Indeed, USA-DDV group is clustered together with the Basque Country and the general Spanish populations, and near to other European samples. In the case of USA-DDA, it is integrated inside the European ancestry cluster, in close proximity to the European descendant population from USA. On the other hand, in concordance with their genetic heterogeneity, Basque Diaspora groups from Argentina are placed between the European ancestry group and the Hispanic and Native American sample sets. Indeed, they are adjacent to the present-day Argentinean population [36] and the geographically-closed Basque Diaspora group from Uruguay [17].

**Fig 4:**
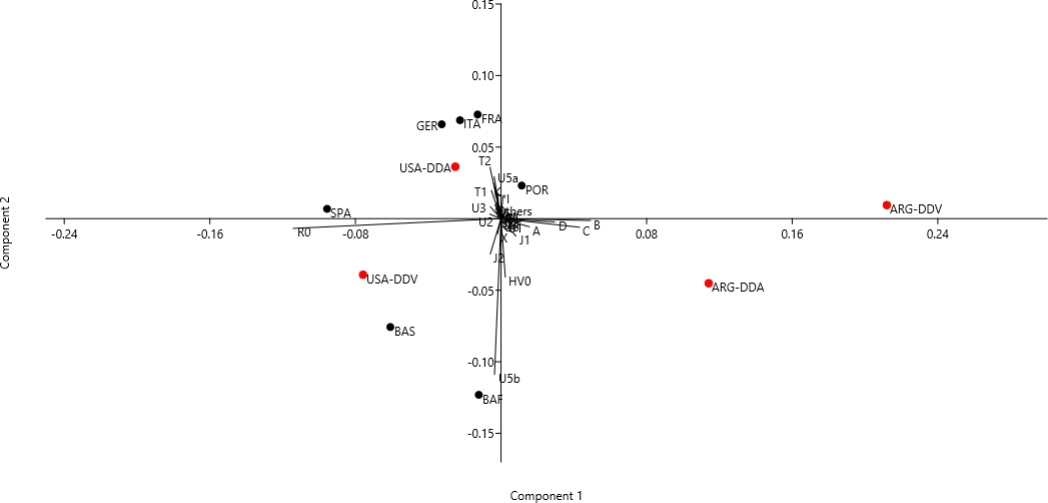
PCA based on mtDNA haplogroup frequencies in Basque Diaspora and European populations. Haplogroups and subhaplogroups included in the PCA biplot are R0 (including H subhaplogroups), HV0, J*, J1, J2, T*, T1, T2, U*, U1, U2, U3, U4, U5, U5a, U5b, U6, U7, U8, K, I, X, W, N, M, L, A, B, C, D, and others. Populations included are as follows: Basque Diaspora populations (in red) from USA (USA-DDV and USA-DDA) and Argentina (ARG-DDV and ARG-DDA), Basque populations from Spain (BAS) and France (BAF),France (FRA), Germany (GER), Italy (ITA), Portugal (POR), and Spain (non-Basque included) (SPA).

Additionally, the PCA allowed a further understanding of the European contribution to the Basque Diaspora populations. The result shown in Fig 4 accounted for 76.78% of the total variation (Component 1: 50.66% and Component 2: 26.12%). Interestingly, the Basque Diaspora groups appeared segregated in distinct quadrants of the PCA representation. Indeed, USA-DDV was placed in the third quadrant with the European Basque populations; actually it was closer to the Spanish Basque population as they shared a high percentage of R0 and U5b, as well as a noticeable frequency of J and HV0 lineages. Besides, the USA-DDA group was near to other European populations, in the second quadrant. Certainly, mtDNA contribution to Diaspora groups from other regions from Europe, and more concretely from Spain, is expected since they have also been historical sources of immigration to America, and consequently could have mixed up with the Basque descendants. Regarding the general Spanish population, it is located near the two USA Diaspora groups, as they shared a certain similarity in the haplogroup pattern. On the other hand, in the Diaspora groups from Argentina, the presence of Native American lineages (A, B, C, and D) and a minor frequency of R0, in comparison with most European populations, conditioned their relatively distant location in the European context.

## Discussion

The Basque Diaspora in the Western USA and Argentina represents populations that have maintained strong cultural and social Basque roots. They settled in a completely different geographic context, consequently providing an outstanding opportunity in order to study the dynamics of Diaspora populations.

Furthermore, the prior differentiation of two different groups in each population, one composed by individuals whose maternal ancestor surnames were Basques (DDV), and the other one, by individuals who descend from Basques but have not maintained their maternal Basque surnames (DDA), has allowed a deeper understanding of the genetic results of the present study. In this regard, non-admixed surname groups constitute useful populations in order to assess the presence of founder effects by comparison to their European Basque source population, whereas admixed surname groups allow us to determine the degree of genetic introgression of American lineages.

Interestingly, mtDNA analysis highlighted a similar degree of haplogroup diversity between DDV groups from Western USA and Argentina and the source Basque populations, regarding to their European maternal input. Therefore, non-important founder effects would have occurred in these Diaspora groups.

The determination of the mtDNA haplogroups in the Diaspora populations has allowed us to discern the origin of their maternal gene pool. As expected, the European ancestry was predominant in the lineages observed in the Diaspora groups. More concretely, the maternal contribution from the Basque source population has been stated in these groups, as it is pointed out by the presence of autochthonous Basque haplogroups such as U5b1f1a and J1c5c1, but in different degree. In this regard, the DDV group in Western USA was the closest group to the European Basque populations, as it can be seen in the MDS plot. Accordingly, no genetic substructure was observed between these populations (p≥0.05). Contrarily, the preservation from the maternal European Basque input in the DDA group in Western USA as well as the two Argentinean Diaspora groups has been lower, as these groups displayed significant differentiation from the source Basque population (p<0.05). In fact, these groups presented a tryhibrid ancestry, with a major European component, followed by Native American and, in a minor proportion, African lineages. Consequently, the introgression of lineages from the local populations would have contributed to differentiate these groups, along with the presence of lineages coming from other immigrants from different regions of Spain, France or the rest of Europe, who likely mixed with Basque descendants. This result is in agreement with a previous study, based on X-STRs, reporting high genetic contribution of host or local populations in these admixed Basque Diaspora groups in Western USA and Argentina, which may have aroused the differentiation from the founder population [15].The genetic results are clearly interrelated with the different geographic, linguistic, and cultural context where the studied Basque Diaspora populations settled down. In Western USA, linguistic and cultural differences would have limited the integration of the newcomers, who would have remained mainly confined within the group, for a long time. Furthermore, Basque families maintained a strong ethnic identity, since men could meet potential wives among the women recruited from the Basque homeland, whereas other men, once they were financially established, sent back to Europe for their wives, who joined them in the United States. The present study unveils that the DDV group has mainly preserved their maternal legacy and limited the genetic introgression of host populations. Consistently, the diversity values for this group was significantly lower (H = 0.9765) than in other USA immigrant populations, as Hispanic-Americans (H = 0.9980) [37] or Afro-Americans (H = 0.9985) [38], which presented a higher admixture degree. Accordingly, a previous study based on Y-STRs also pointed out that no significant differences in paternal lineages were found among Basque Diaspora group in Western USA and the autochthonous European Basque population [14]. Consequently, neither geographical separation from their original homeland nor the contact with the American population seems to have caused a significant impact on the female or male genetic legacy of the Basque Diaspora population in Western USA. Contrastingly, in the case of Argentinean Basque Diaspora the lack of linguistic barriers probably promoted a widespread immigration from the Basque Country. Furthermore, the broad integration of the newcomers in the host territory would have facilitated their admixture with other local groups, thereby incorporating their lineages in the genetic pool. This is evidenced by the genetic proximity between the host Argentinean population and the Diaspora DDV group, as it is shown in the MDS plot. Thereafter, the characteristics of the immigration in Argentina involving mainly male migrants and the lack of impeding factors for their integration might have favored the mating between Basque men and non-Basque women, thus partly explaining the non-European contribution to the genetic legacy. This sexual asymmetry is a common feature in admixed populations in the American continent [39–41] and it has also been observed in another Basque Diaspora group located in Uruguay (Trinidad Region), which showed a high mtDNA diversity suggesting that the newcomers did not remain isolated but intermarried with other groups in Uruguay [17].

Additionally, the heterogeneity of haplogroups in the Diaspora groups in Argentina is in accordance with the diversity observed in the present-day multi-ethnic population [36]. In this regard, the Native American component has been described as remarkably strong in Argentinean admixed groups, ranging from 41–70%. Moreover, although the impact of the presence of African slaves brought to Argentina by Europeans during colonization was low compared to other regions from America, their genetic contribution has also been detected (1–3%).

The data from the present study has also revealed that no genetic substructure existed among the two Basque Diaspora groups in Western USA, neither into the Argentinean Diaspora groups (p>0.05). However, the North-American Diaspora groups are clearly differentiated from the Argentinean Diaspora groups, as well as from their corresponding host populations. This genetic stratification may have important implications in Forensic Genetics and Genetic Epidemiology. On the one hand, forensic studies cannot ignore the population substructure, as the evaluation of the weight of the evidence can be biased [42]. Hence, the present study provides specific databases for the Diaspora groups, since the application of an mtDNA general database from the host population would not be accurate for these groups in forensic casework match probability assessments. On the other hand, the knowledge of the genetic structure of the target population in medical studies is necessary to avoid wrong associations among certain populations and diseases [43, 44]. The study of Diaspora populations in epidemiology studies is a matter of great interest, insofar as they can potentially constitute suitable models in order to analyze the effect of different environmental, dietary, or lifestyle factors in genetic susceptibility to diseases, in comparison with the source population. These populations supposedly share a common genetic background with the source population; therefore, the genetic component of the etiology susceptibility would not constitute an additional variable, being the differential environment the main key factor. The present study has inferred that the Basque DDV group in Western USA has actually maintained the maternal genetic legacy from the Basque source population in Europe. Consequently, the study of this Basque group in Western USA and the European Basque populations constitutes a suitable model for epidemiology research as they share a common genetic component but different environmental, dietary or lifestyle influences. Furthermore, the possibility to study the admixed population of Basque descendants in Western USA provides us with another control group of interest. By contrast, the study of the Argentinean Diaspora groups has revealed that nowadays they constitute differentiated groups from the source populations and, consequently, the genetic component constitute another important variable which will have to be take into account in order to compare these groups for epidemiological purposes. Thereby, the assessment of the genetic structure of a population is necessary for a precise and accurate understanding of the results in forensics, as well as, in associative genetics studies.

## Supporting Information

S1 Table Mitochondrial DNA haplogroup data used for phylogenetic comparisons. For data retrieved from GenBank database, GenBank accession numbers are given.
(XLSX)

S2 Table Haplotypes of Basque Diaspora populations in Western USA (USA-DDV and USA-DDA) and Basque Diaspora populations in Argentina (ARG-DDV and ARG-DDA). Range included: 16024–576.
(XLSX)

S3 Table Haplotypes of Basque Diaspora populations shared with Basque populations from Spain and France. The relative frequency (%) of each haplotype is indicated.
(XLSX)

S4 Table Haplogroup frequencies in Basque Diaspora populations in Western USA (USA-DDV and USA-DDA) and Basque Diaspora populations in Argentina (ARG-DDV and ARG-DDA). (XLSX)
